# Difficult intubation in a patient with acute epiglottitis and abscess complicated with cervical necrotizing fasciitis: A case report

**DOI:** 10.1097/MD.0000000000038658

**Published:** 2024-06-21

**Authors:** Guanghua Fu, Luyao Yang, Guisheng Wu

**Affiliations:** a Department of Anesthesiology, Liaocheng People’s Hospital, Liaocheng, Shandong, China.

**Keywords:** acute epiglottitis, anesthesia, case report, cervical necrotizing fasciitis, difficult airway, neck abscess

## Abstract

**Introduction:**

Acute epiglottitis is not uncommon and it can cause high mortality due to airway obstruction. Acute epiglottitis complicated with cervical necrotizing fasciitis has rarely been reported, and it is also a life-threatening disease with a fatality rate of 7% to 50%.

**Patient concerns:**

A 64-year-old woman presented to our hospital with chief complaints of sore throat and cervical swelling, long with foreign body sensation and hoarseness. Endoscopic laryngoscopy showed erythematous and swollen epiglottis with purulent secretions on the surface. Computed tomography (CT) scan showed swollen epiglottis and swelling of the neck with air- and fluid-containing necrotizing tissue.

**Diagnoses:**

The diagnosis was acute epiglottitis and abscess complicated with cervical necrotizing fasciitis.

**Interventions:**

With the patient in awake condition, airway access was established by performing intubation with adjunctive use of gum elastic bougie, followed by surgical debridement under general anesthesia; a flap was used for skin coverage and intravenous piperacillin-tazobactam was administered.

**Outcomes:**

The patient was discharged without complications.

**Conclusion:**

Gum elastic bougie is a usable tool in difficult intubation. Adequate pre-anesthesia evaluation, patient sedation, and gentle manipulation assured the intubation success in this case.

## 1. Introduction

Acute epiglottitis is not an uncommon disease, and it can cause high mortality due to airway obstruction.^[1]^ Reportedly, <10% of cases of acute epiglottitis require airway intervention.^[2]^

Cervical necrotizing fasciitis is a rare, rapidly progressive, and life-threatening disease, with a fatality rate of 7% to 50%.^[3]^ It is often caused by the direct spread of dental infection. However, cases of cervical necrotizing fasciitis as a complication of acute epiglottitis have rarely been reported.^[4]^

Tracheal intubation may be accepted as the definitive intervention for critically ill patients with airway compromise.^[5]^ According to the American Society of Anesthesiologists guidelines, a difficult airway is defined as a clinical situation in which a conventionally trained anesthesiologist experiences difficulty with facemask ventilation of the upper airway, difficulty with tracheal intubation, or both.^[6]^

Herein, we report a case of acute epiglottitis and abscess complicated with cervical necrotizing fasciitis, in which airway access was obtained through intubation with adjunctive use of gum elastic bougie. The patient was successfully cured following surgical debridement and antibiotic therapy.

## 2. Case presentation

A 64-year-old woman was admitted to our hospital with the chief complaints of sore throat and cervical swelling for 2 days. The associated symptoms included foreign body sensation and hoarseness. Initially, she was administered intravenous antibiotic therapy at a local clinic, but her symptoms did not resolve. The next morning, she came to our hospital with severe odynophagia. Her past history was unremarkable except for a history of hypertension.

**Figure 1.**
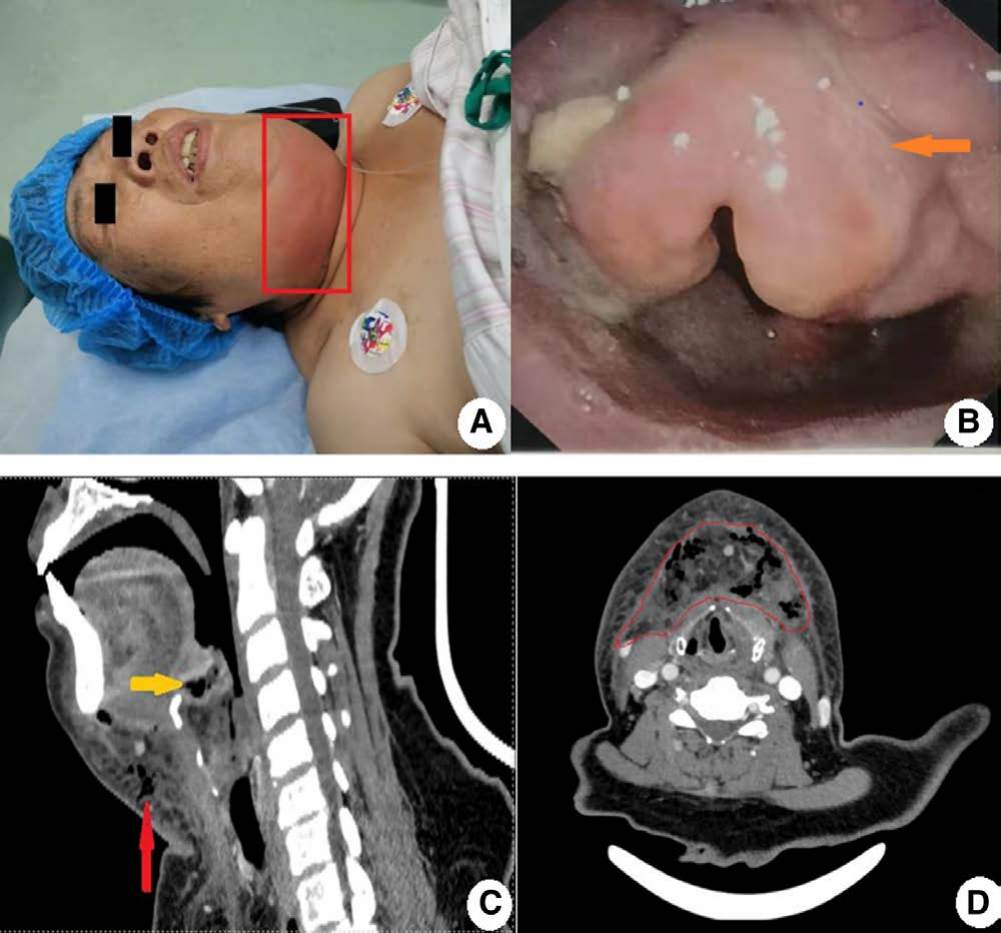
(A) The patient was an old, obese woman with an erythematous and swollen neck. (B) Endoscopic laryngoscopy showed hoof-like, erythematous, and swollen epiglottis with purulent secretions on its surface (orange arrow). (C) Cervical CT scan (sagittal section) showed a swollen epiglottis with abscess cavity (yellow arrow); the air-containing necrotizing tissue in the neck is indicated by the red arrow. (D) Horizontal view of cervical CT scan showing necrotizing tissue in the neck with air and abscess cavity (red area). CT = computed tomography.

On examination, she was 160 cm in height, 70 kg in weight, and her body mass index was 27.3 kg/m^2^. On physical examination, she was febrile (38.2°C) and had tachycardia (heart rate 110 bpm) with normal blood pressure (117/92 mm Hg). Her neck was short and thick. Physical examination revealed a tender, diffuse erythematous swelling with crepitation in the whole anterior neck (Fig. 1A). Electronic laryngoscopy showed a swollen epiglottis with purulent secretions on the right surface, while the glottis was patent (Fig. 1B).

Laboratory investigations showed elevated white blood cell count (19.39 × 10^9^/L) and C-reactive protein level (114.62 mg/L). Her blood glucose was 8.21 mmol/L, and the glycosylated hemoglobin (HbA1c) level was 6.4%. A cervical computed tomography (CT) scan showed a markedly enlarged epiglottis with an abscess cavity formed inside; in addition, in the submentum and the left neck, there was an ill-defined irregular soft tissue density shadow (approximately 20 × 12 cm) containing multiple gas shadows (Fig. 1C and D).

The patient was diagnosed with acute epiglottis and abscess combined with cervical necrotizing fasciitis. She was immediately started on intravenous antibiotics (mezlocillin sodium 2 g, qd) and steroid (methylprednisolone sodium succinate 80 mg, qd) therapy, but her condition did not improve. On the third day, she developed dyspnea and her pulse blood oxygen saturation (SPO_2_) decreased to 93%. Obtaining airway access was essential and we planned for tracheotomy followed by surgical debridement.

On pre-anesthetic evaluation, she showed restricted neck movement. The mouth opening was only 1 finger, and her Mallampati score was class IV.^[7]^ Given the severe neck swelling and her poor general condition, we attempted intratracheal intubation.

**Figure 2.**
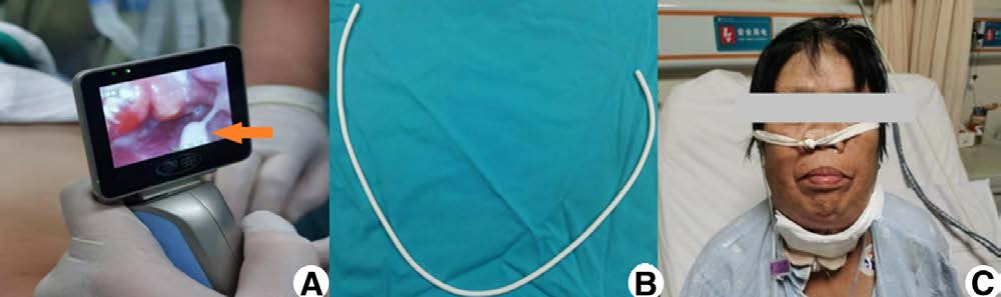
(A) Video laryngoscopy photograph showing a severely swollen epiglottis and lack of visualization of glottis. The white equipment on the right lower part is the headend of the gum elastic bougie (orange arrow). (B) Gum elastic bougie: a 60-cm-long tracheal tube introducer fabricated from a braided polyester base with a resin coating and a smooth angled distal tip. (C) Postoperative photo showing the skin of the patient neck covered with a flap after surgical debridement and a prophylactic tracheotomy.

The intratracheal intubation was done while the patient was awake with mask ventilation. Before intubating, sufentanil 10 µg, methylprednisolone 40 mg, penehyclidine hydrochloride 0.5 mg, and dexmedetomidine 70 µg were administered intravenously, and 2% lidocaine 5 mL was gargled twice by the patient for surface anesthesia of oropharynx. On the first trial to intubate, video laryngoscopy showed a severely swollen hoof-like epiglottis, the glottis was not visualized, and the scope classification was grade III (Fig. 2A).^[8]^ Then, 2% lidocaine 5 mL was sprayed over the trachea for surface anesthesia, while the patient was asked to cough in order to augment the effect. On the second trial, a gum elastic bougie (Fig. 2B) was directed just posterior to the epiglottis, and a click sensation confirmed its placement in the trachea; subsequently, a 6.5 mm endotracheal tube was successfully advanced into the trachea. After the airway was accessed, surgical debridement was performed under general anesthesia. A 5 cm horizontal incision was made in the submentum and the abscess cavity seen in the oral floor and submentum was dissected. The purulent secretions in the cavity were removed and the cavity was irrigated with hydrogen peroxide and saline solution. Then, a drainage tube was placed and the flap was used for skin coverage. The culture of debrided tissue showed Gram-positive cocci (+) and Gram-negative bacilli (++). Prophylactic tracheotomy was performed and a 7.5 mm endotracheal tube was placed just before the completion of surgery (Fig. 2C).

After surgery, the patient was treated with intravenous piperacillin-tazobactam (3.375 mg every 6 hours for 4 days and then 3.375 mg every 8 hours for 9 days) as well as operative cavity irrigation with hydrogen peroxide and tinidazole. Her symptoms resolved gradually and the endotracheal tube was removed on day 42 after surgery. On day 45, the patient was discharged without any complications and continued to be well on serial follow-up visits.

## 3. Discussion

Approximately 24% of patients with acute epiglottitis may develop epiglottic abscess.^[9]^ Cervical necrotizing fasciitis was first described by Melency in 1924 as a severe, rapidly progressive disease that can cause potentially fatal complications such as airway obstruction, pneumonia, pulmonary abscess, mediastinitis, jugular venous thrombophlebitis, and septic shock.^[4]^ Cervical necrotizing fasciitis is usually caused by dental diseases or trauma, and in rare cases is complicated by acute epiglottis.^[10]^

In this study, we reported a case of acute epiglottitis and abscess complicated with cervical necrotizing fasciitis. To the best of our knowledge, no similar case has been reported before. Gollapalli et al^[10]^ reported a case of acute epiglottitis complicated with cervical necrotizing fasciitis, and they cured the patient through conservative surgical treatment, including incision, drainage, and antibiotic therapy. Compared to our patient, their patient did not have epiglottic abscess or suffer dyspnea, so airway intervention was not done. Komasawa et al^[11]^ reported a case of acute epiglottitis with abscess complicated with severe deep neck abscess. Similar to our case, they confronted the problem of difficult airway management. They successfully used a new instrument, Pentax-AWS Airwayscope, and a soft-tipped endotracheal tube exchanger to assist intubation. In our case, we used gum elastic bougie to open the airway in order to facilitate intubation, which is widely available. Besides, Rongming^[3]^ and Kraus^[4]^ also reported cases of acute epiglottitis and cervical necrotizing fasciitis. However, in their cases, the diseases descended to involve the chest, causing mediastinal necrotizing fasciitis or abscess, resulting in a more severe condition compared to our case.

Cervical necrotizing fasciitis is an infectious disease of the fascial planes of the neck, which can cause extensive liquefaction, necrosis, and suppuration of the deep cervical fascia.^[3]^ It has rarely been reported to occur as a complication of tonsillitis, peritonsillar abscess, and acute epiglottitis.^[8,10]^ The present case responded poorly to conservative treatment with antibiotics or steroids and developed dyspnea with a decrease in SPO_2_, which was a sign of airway compromise. According to the studies by Pineau^[1]^ and Katori,^[8]^ the occurrence of dyspnea and a rapidly progressive clinical course in acute epiglottitis necessitates airway intervention. Therefore, gaining airway access was crucial for our patients. Regarding the choice of method for airway management, Felton et al recommended that awake intubation can replace traditionally supported surgical airway intervention.^[12]^ Thus we opted for awake intubation to gain airway access. Another factor affecting the choice was the swollen neck and extensive necrotizing infection.

However, the patient had several factors that predicted difficult intubation, including obesity, limitation of cervical movement, difficulty in mouth opening, and airway obstruction due to swollen epiglottis.^[2,13]^ We used a gum elastic bougie as an adjunct equipment for intubation. The first reported use of gum elastic bougie was by MacIntosh in 1949^[14]^ and it has subsequently been widely used in European countries in case of difficult tracheal intubation.^[15,16]^ Jabre et al recommended using gum elastic bougie as the first strategy in case of difficult intubation because the device is simple and easy to use.^[15]^ However, in the study by Shah et al, the success rate of using gum elastic bougie in difficult intubation was 73.7%, and they found that the characteristics of “palpable clicks” and “holdup” were unreliable.^[16]^ In the present case, gum elastic bougie was successfully used for assisting the intubation, and in the process, we felt “palpable clicks” as a sign of its position inside the trachea. We believe that gum elastic bougie is a useful equipment in difficult intubation.

Besides, we administered sedation and analgesia before conducting anesthesia to reduce the patient restlessness and decrease the risk of suffocation due to aggravation of edema of the soft airway tissue. Moreover, the intubation was performed by an experienced anesthetist with gentle manipulation.

Surgical debridement is often necessary in the treatment of cervical necrotizing fasciitis to control the spread of infection. In this case, we did not use high-grade antibiotics as that choosing the antibiotic based on the antimicrobial susceptibility test might be more important. Thus, early diagnosis, radical surgical debridement, and targeted intravenous antibiotic therapy can cure this disease.

## 4. Conclusions

We report a rare case of acute epiglottitis and abscess complicated with cervical necrotizing fasciitis. The patient was cured by airway intervention with the assistance of gum elastic bougie, surgical debridement, and antibiotic therapy.

## Author contributions

**Conceptualization:** Guanghua Fu.

**Formal analysis:** Luyao Yang.

**Funding acquisition:** Guisheng Wu.

**Investigation:** Guanghua Fu.

**Resources:** Luyao Yang.

**Software:** Guisheng Wu.

**Supervision:** Guanghua Fu.

**Visualization:** Luyao Yang, Guisheng Wu.

**Writing – original draft:** Guanghua Fu, Luyao Yang, Guisheng Wu.

## References

[1] Pineau PM Gautier J Pineau A. Intubation decision criteria in adult epiglottitis. Eur Ann Otorhinolaryngol Head Neck Dis. 2021;138:329–32. 33358682 10.1016/j.anorl.2020.12.001

[2] Tapiovaara LK Aro KLS Bäck LJJ. Comparison of intubation and tracheotomy in adult patients with acute epiglottitis or supraglottitis. Eur Arch Otorhinolaryngol. 2019;276:3173–7. 31489494 10.1007/s00405-019-05624-0 PMC6811371

[3] Ge R Mao Y Zhang XL. Cervical necrotizing fasciitis and a descending mediastinal abscess caused by acute epiglottitis with diabetes mellitus: a life-threatening complication. Diabetes Res Clin Pract. 2012;95:e31–33. 22088790 10.1016/j.diabres.2011.10.010

[4] Kraus M Gatot A Leiberman A. Acute necrotizing epiglottitis resulting in necrotizing fasciitis of the neck and chest. Otolaryngol Head Neck Surg. 2001;124:700–1. 11391269 10.1177/019459980112400624

[5] Morton T Brady S Clancy M. Difficult airway equipment in English emergency departments. Anaesthesia. 2000;55:485–8. 10792145 10.1046/j.1365-2044.2000.01362.x

[6] Apfelbaum JL Hagberg CA Caplan RA. Practice guidelines for management of the difficult airway: an updated report by the American Society of Anesthesiologists Task Force on Management of the Difficult Airway. Anesthesiology. 2013;118:251–70. 23364566 10.1097/ALN.0b013e31827773b2

[7] Stutz EW Rondeau B. Mallampati Score. 2023 Aug 5. In: StatPearls. Treasure Island (FL): StatPearls Publishing; 2024 36256766

[8] Katori H Tsukuda M. Acute epiglottitis: analysis of factors associated with airway intervention. J Laryngol Otol. 2005;119:967–72. 16354360 10.1258/002221505775010823

[9] Hindy J Novoa R Slovik Y. Epiglottic abscess as a complication of acute epiglottitis. Am J Otolaryngol. 2013;34:362–5. 23391346 10.1016/j.amjoto.2013.01.003

[10] Gollapalli RB Naiman AN Merry D. Cervical necrotizing fasciitis as a complication of acute epiglottitis managed with minimally aggressive surgical intervention: case report. Ear Nose Throat J. 2015;94:E5–7. 26214679 10.1177/014556131509400714

[11] Komasawa N Minami T. Difficult airway management in a patient with combined severe deep neck abscess and acute epiglottitis with abscess. J Clin Anesth. 2014;26:581. 25439424 10.1016/j.jclinane.2014.05.003

[12] Felton P Lutfy-Clayton L Smith LG. A retrospective cohort study of acute epiglottitis in adults. West J Emerg Med. 2021;22:1326–34. 34787558 10.5811/westjem.2021.8.52657 PMC8597686

[13] Bazurro S Ball L Pelosi P. Perioperative management of obese patient. Curr Opin Crit Care. 2018;24:560–7. 30299311 10.1097/MCC.0000000000000555

[14] MacIntosh RR. An aid to oral intubation. Br Med J. 1949;1:28.

[15] Jabre P Combes X Leroux B. Use of gum elastic bougie for prehospital difficult intubation. Am J Emerg Med. 2005;23:552–5. 16032630 10.1016/j.ajem.2004.12.005

[16] Shah KH Kwong BM Hazan A. Success of the gum elastic bougie as a rescue airway in the emergency department. J Emerg Med. 2011;40:1–6. 18996675 10.1016/j.jemermed.2008.04.045

